# Sheng-ji Hua-yu formula promotes diabetic wound healing of re-epithelization via Activin/Follistatin regulation

**DOI:** 10.1186/s12906-017-2074-8

**Published:** 2018-01-29

**Authors:** Le Kuai, Jing-ting Zhang, Yu Deng, Shun Xu, Xun-zhe Xu, Min-feng Wu, Dong-jie Guo, Yu Chen, Ren-jie Wu, Xing-qiang Zhao, Hua Nian, Bin Li, Fu-lun Li

**Affiliations:** 1 Department of Dermatology of Yueyang Hospital, Shanghai University of TCM, Shanghai, 200437 China; 2 Department of Burns and Plastic Surgery, The Seventh People’s Hospital of Integrated Traditional Chinese and Western Medicine, affiliated with Shanghai University of Traditional Chinese Medicine, Shanghai, 200137 China; 3 Pharmaceutical Center of Yueyang Hospital, Shanghai University of TCM, Shanghai, 200437 China; 4 School of Medicine, Chengdu University, Chengdu, 610106 China

**Keywords:** Chinese herbal, Diabetic ulcer, Wound healing, Inflammation, Re-epithelization, Activin, Follistatin

## Abstract

**Background:**

Sheng-ji Hua-yu(SJHY) formula is one of the most useful Traditional Chinese medicine (TCM) in the treatment of the delayed diabetic wound. However, elucidating the related molecular biological mechanism of how the SJHY Formula affects excessive inflammation in the process of re-epithelialization of diabetic wound healing is a task urgently needed to be fulfilled. The objectives of this study is to evaluate the effect of antagonisic expression of pro−/anti-inflammatory factors on transforming growth factor-β(TGF-β) superfamily (activin and follistatin) in the process of re-epithelialization of diabetic wound healing in vivo, and to characterize the involvement of the activin/follistatin protein expression regulation, phospho-Smad (pSmad2), and Nuclear factor kappa B p50 (NF-kB) p50 in the diabetic wound healing effects of SJHY formula.

**Methods:**

SJHY Formula was prepared by pharmaceutical preparation room of Yueyang Hospital of Integrated Traditional Chinese and Western Medicine. Diabetic wound healing activity was evaluated by circular excision wound models. Wound healing activity was examined by macroscopic evaluation. Activin/follistatin expression regulation, protein expression of pSmad2 and NF-kB p50 in skin tissue of wounds were analyzed by Real Time PCR, Western blot, immunohistochemistry and hematoxylin and eosin (H&E) staining.

**Results:**

Macroscopic evaluation analysis showed that wound healing of diabetic mice was delayed, and SJHY Formula accelerated wound healing time of diabetic mice. Real Time PCR analysis showed higher mRNA expression of activin/follistatin in diabetic delayed wound versus the wound in normal mice. Western Blot immunoassay analysis showed reduction of activin/follistatin proteins levels by SJHY Formula treatment 15 days after injury. Immunohistochemistry investigated the reduction of pSmad2 and NF-kB p50 nuclear staining in the epidermis of diabetic SJHY versus diabetic control mice on day 15 after wounding. H&E staining revealed that SJHY Formula accelerated re-epithelialization of diabetic wound healing.

**Conclusion:**

The present study found that diabetic delayed wound healing time is closely related to the high expression level of activin/follistatin, which leads to excessive inflammation in the process of re-epithelization. SJHY Formula accelerates re-epithelialization and healing time of diabetic wounds through decreasing the high expression of activin/follistatin.

**Graphical abstract:**

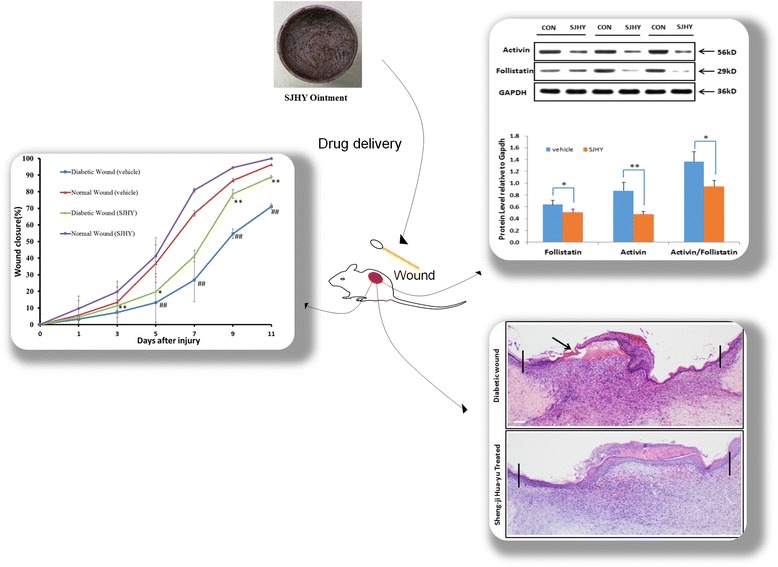

## Background

An estimated 415 million people aged 20–79 were afflicted with diabetes in 2015, and the number is predicted to rise to 642 million by 2040 [1]. Mechanisms for the delayed diabetic wound healing are still poorly understood [2]. China has the largest number of diabetics in the world since 2013 and the rate of increase is much higher than in Europe and the U.S. [3]. Thereby, it is an urgent task to understand the mechanism of diabetic wound healing and find more efficient and safer treatment regimen to control diabetic ulcers.

Wound healing is an orchestrated process consisting of three overlapping phases: inflammation, restoration and maturation [4]. Delayed wound healing in diabetes is related to excessive inflammation [5]. Re-epithelialization, new vessel formation, growth factor production, and immune responses are among those functions altered in diabetic wound healing [2]. Furthermore, recent study has indicated that inflammatory phase in the process of re-epithelialization of diabetic wound healing lasts for an abnormally long time with delayed resolution, and the wound is in a state of prolonged inflammation, slow to transition to the latter two phase of wound healing [6]. Chronic inflammation of diabetic wound healing results not only from an excess of pro-inflammatory cytokines, but also from an excess of anti-inflammatory and healing-associated cytokines. This can be attributed to the imbalanced expression of pro-inflammatory and anti-inflammatory factors [7, 8]. Transforming growth factor-β(TGF-β) is a pivotal inflammatory factor in wound healing and scar formation which many studies have been focused on [9–12]. Activins are members of TGF-β superfamily, and similar to other members of the TGF-β superfamily, they exert their biological effect mainly through the Smad signal pathway by binding to transmembrane receptor serine/threonine kinases receptor (type I and II membrane receptors). Phosphorylated Smad2 and Smad3 bind a co-Smad, Smad4, forming heteromeric Smad complexes that translocate into the nucleus to regulate transcription of target genes, mediating their biological roles including regulation of inflammatory and immunity reaction via NF-kB, TNF-α, IL-1β, IL-6, INOS. During wound healing processes, activin/TGF-β is a known chemokine associated with cell inflammation and tissue repair through the regulation of skin and immune cell migration, proliferation, differentiation, extracellular matrix synthesis and secretion. Activin signaling can regulate re-epithelialization, inflammation and scar formation, which play a critical regulator role in cutaneous wound healing. The action of activin is antagonized by follistatin in vivo and in vitro. Follistatin and activin together compose a balanced system in wound healing processes and scar formation [13].

So far, the mechanisms of action for activin/follistatin in the process of cutaneous wound healing are still not clear. An early study examined the response of activin A and activin B mRNA expression 1 day after skin injury have found high expression levels of activin A and B within the first 7 days after wounding. Expression of activin A mRNA returned to the basal level on day 13 after wounding, whereas high levels of activin B persisted. In situ hybridization studies revealed expression of activin A in the granulation tissue below the wound and activin B in the hyperproliferative epithelium at the wound edge and in the migrating epithelial tongue. Nevertheless, no significant induction of receptor gene expression has been seen during the repair processes [14]. Recent reports have shown that activin B is able to activate c-Jun N-terminal kinase(JNK), extracellular regulated protein kinases(ERK) and Ras homolog gene family,member A (RhoA)-JNK signaling pathways to promote wound healing [15, 16]. Similarly, down-regulation of activin B expression in rats leads to significantly delayed wound healing [17]. Transgenic mice overexpressing activin A in keratinocytes under control of the keratin 14 promoter (Act mice) are characterized with an acceleration of re-epithelialization and skin wound healing time, as well as the excessive granulation tissue formation and scar formation [18]. The above prompts while activin is useful for accelerating wound healing, it is easy to form excessive scar tissue. Transgenic mice overexpressing follistatin inhibit scar formation after injury [19], which implies that up-regulation of follistatin expression can reduce the inflammatory reaction and scar formation in the process of cutaneous wound healing. Therefore, study should not merely focus on activin or follistatin but also to analyze the unbalanced expression between activin and follistatin. Study speculated that activin could initiate the differentiation of epithelial cells by specifically blocking follistatin and its downstream signals. However, subsequent studies have found activin receptors on many different types of cells other than epithelial cells. Thus, how activin/follistatin function affects wound healing is still unclear [20].

Traditional Chinese Medicine(TCM) has a long history as a method to prevent and treat chronic skin ulcer (CSU). Preliminary studies have found herbal Formula treatment to be efficient in the management of diabetic skin ulcers, and wound healing time is 2–3 days less than in the patient group treated using conventional western medicine (WM) [21]. The mechanism of action might be related to the inhibition of wingless/int1-related(Wnt) signaling pathway, which partly illuminates the molecular biology mechanism for herbal Formula treatment, setting up a firm basis for clinical work [21]. Sheng-ji Hua-yu(SJHY) formula is one of the most effective TCM in the treatment of the delayed diabetic wound. Moreover, studies have shown that SJHY Formula increases the level of collagen types I and III in granulation tissue of rats on the seventh day of wound healing [22], regulating the ratio of collagen type I and III and adjusting their metabolism in wound healing fibroblasts [23], as well as regulating the expression of matrix metalloprotease-3(MMP-3) and tissue inhibitor of metalloproteinase-1(TIMP-1) in ulcer tissues of diabetic rats [24]. Yet reaction mechanisms for excessive inflammation in the process of re-epithelialization is not clear yet.

Based on these evidence, the trial was designed to evaluate the effect of activin/follistatin regulation in the process of re-epithelialization of diabetic wound healing in vivo and elucidate the involvement of activin/follistatin protein expression regulation, pSmad2, and NF-kB p50 in the diabetic wound healing effects of SJHY formula.

## Methods

### Plant material

Drug Preparation. SJHY Formula was comprised of 8 Chinese herbs, as showed in Table 1. The dosage used in the present study was determined according to the Chinese Pharmacopoeia(2015 edition), made by the pharmacy department of Yueyang Hospital of Intergrated Traditional Chinese and Western Medicine, Shanghai University of Traditional Chinese Medicine. (1) Chinese herbs were extracted with 1500 ml 95% ethanol by maceration for an hour. The extract was concentrated in a rotary evaporator. The extracting operation was repeated. (2) Two parts of extract was mixed and let sit, filtered and concentrated to 175 ml. (3) 12.5 ml of the extract was further concentrated to 7 ml, which was then mixed with carbomer. (4) The pH value of the mixture was regulated between 6 and 8 by using triethanolamine as neutralizer. (5) The mixture was stored at 4 °C.

**Table 1:** Ingredients of SJHY Formula Ointment with English Translations

Main composition	Latin scientific name	Plant part (s)	Amount (g)
Radix Astragali	*Astragalus membranceus (Fisch) Bunge*	Radix	60.05
Radix Salviae Miltiorrhizae	*Salvia miltiorrhiza Bunge*	Rhizoma	15.03
Radix et Rhizoma Rhei	*Rheum palmatum L.*	Rhizoma	15.12
Resina Draconis	*Daemonorops draco (Willd.) Blume*	Resin	10.06
Radix Lithospermi	*Arnebia euchroma (Royle) I.M. Johnst.*	Radix	30.16
Angelica dahurica	*Angelica dahurica (Hoffm.) Benth. & Hook.f. ex*	Radix	30.35
	*Franch. & Sav.*		
Nacre	*Hyriopsis cumingii (Lea)*	–	30.10
Calamine	*Calamine*	–	30.05

### Quantitative analysis of SJHY formula by HPLC

The SJHY Formula was analyzed by liquid chromatograph-mass spectrometer (LC-MS) using Agilent 1200 Series analytical systems equipped with a photodiode array (PDA) detector combined with a 6130 Series ESI mass spectrometer. Briefly, constant-weight 3.65 mg loureirin A and 3.62 mg loureirin B were dissolved in the 10 ml measuring cylinder with methanol, diluted to scale mark, shook well as the reference solutions required. The next step was to dissolve 1 g SJHY Formula with methanol-diethyl ether(5:95) to 20 ml, heated in the water bath (60 °C) for 5 min, cooling then taking only the supernatant. Methanol-diethyl ether(5:95)10 ml was added to residue, which was then extracted as the former method. After five more times of above extraction, the six supernatant were merged and dried in a water bath. Residue was dissolved with methanol, which was then mixed to 5 ml in a measuring cylinder. The solution was filtered using 0.45 μm membrane before injection.

### Chemicals, reagents

Streptozotocin, Sigma Co.,Ltd.; Carbomer, Animal experiment center, Shanghai University of Traditional Chinese Medicine; High-fat diet and normal diet, Shanghai Pu Lu Teng Biological Technology Co.,Ltd.; Isoflurane, Animal experiment center, Shanghai University of Traditional Chinese Medicine; iTaqUniversal SYBR Green Supermix, Bio-Rad Co.,Ltd.; micro centrifuge, TIANGEN Biotech(Beijing) Co.,Ltd.; Lab-dancer, Beijing Zhong Yi Huifeng technology Co.,Ltd.; PCR Thermocycle Instrument, Roche Diagnostics GmbH; Qubit, Invitrogen Co.,Ltd.; Heraeussepatech,Thermo Co.,Ltd.; ACCU-CHEKACTIVE Glucometer and test strips, Roche Diagnostics GmbH; anesthesia machine, Animal experiment center, Shanghai University of Traditional Chinese Medicine; Olympus E-620 Digital Camera, Olympus Co.,Ltd.; ImageJ 1.42q Software, National Institutes of Health; Quick wound healing adhesive plaster, Smith & Nephew; HE Staining Kit, Shanghai Beyotime Biotechnology Co.,Ltd.; Cell Lysis Buffer, Cell Signaling Technology Co.,Ltd.; Small protein electrophoresis and transfer printing slot Tetra Protein, Bio-Rad Co.,Ltd.; ECL chemiluminescence detection kit, Santa Co.,Ltd.; Odyssey quantitative fluorescence scanner, IL-COR Co.,Ltd.; IHC KIT, Santa Co.,Ltd.; Trypsin Induced Antigen Retrieval Solution, sh-genmed Co.,Ltd.; DK-8D thermostatic water tank, Shanghai Jing Hong Laboratory Instrument Co.,Ltd.; Olympus BH2 upright metallurgical microscope, Olympus Co.,Ltd.

### Animals

Two hundred thirty female C57BL/6 (8 weeks old) bred in the Laboratory Animal Center of Shanghai University of TCM. The animals were kept under standard temperature (23 ± 2 °C), in specific pathogen-free animal(SPF) grade cages under aseptic conditions and had high-fat or standard diet and water ad libitum. The animals were randomly divided into normal group and diabetic group. The former group had 96 mice and the latter group had 134 mice. The animals were obtained from Shanghai Slac Laboratory Animal Co., Ltd. (scxk Shanghai 2012–0002) The high-fat and standard diet was produced by Shanghai Pu Lu Tong Biological technology Co.,Ltd.

### Diabetic animal model

The mice of the diabetic group were fed with a high-fat diet (consist of 54.6% normal diet,16.9% lard, 14% sucrose, 10.2% casein, 2.1% premix and 2.2% maltodextrin; Percentage of energy: crude protein 20 g/100 g, fat 40 g/100 g, carbohydrates 40 g/100 g.) for 4 weeks. Streptozotocin(stz) was dissolved in 0.1 M sodium citrate buffer solution (pH 4.5). All mice except the normal vehicle group took 2% streptozotocin (100 mg kg^−1^) citrate solution 0.2 ml i.p. every other day, twice after ambrosia for 12 h. The mice given 2% streptozotocin citrate solution had access to 5% Glucose solution 4 h later [25]. After 7 days, blood glucose levels were measured and mice with glycemia ranging between 279 mg dl^−1^ (minimum level) and 460.8 mg dl^−1^ (maximum level). The diabetic model would be considered successful if blood glucose concentration was higher than 300.6 mg dl^−1^ and diabetic mice were monitored for polyuria, polyphagia, polydipsia, and glycemia. Mice manifesting a body weight loss above 20% or a poor motility were excluded. Body weight of diabetic mice were measured: (average values ± s.d.): 20.38 ± 1.78 g (before treatment); 18.74 ± 1.26 g(1 week after streptozotocin); 18.56 ± 1.19 g(end of the experiment). 96 successfully established diabetic mice were used (average glycemic levels: 401.4 mg dl^−1^), and randomly divided into diabetic vehicle group and diabetic SJHY-treated group. Normal group mice were randomly divided into normal vehicle group and normal SJHY-treated group. Each group was monitored on day 1, 3, 5, 7, 11, 15, 8 mice at every time point.

### Wound model

Three days after experimentation, mice were anaesthetized with isoflurane. The skin on both sides of the spine was shaved 4 cm prior to the experiment. Four 6 mm-wide-circle, 2 mm-deep wounds sized by circular metal punch, were made under aseptic conditions on the mice, and left undressed to the open environment [26].

### Treatments

For diabetic SJHY-treated group and normal SJHY-treated group: The treatments were applied with SJHY ointment (dose-0.5 g/cm^2^/day) immediately after punch and every 24 h till the wounds were completely healed, respectively.

For diabetic vehicle group and normal vehicle group: the wounds were treated with topical application of carbomer (dose-0.5 g/cm^2^/day) to the mice every 24 h till the wounds were completely healed, respectively.

The wounds were cleaned every day by normal saline(NS) before the treatment.

### Macroscopic evaluation

Vein blood from tail of mice was drawn on day 1, 3, 5, 7, 9,11. Blood glucose level was measured with Roche ACCU-CHEK ACTIVE Glucometer. Meanwhile, the measurement of the wound area was performed as follows: the quick wound healing adhesive plaster was pressed to the ulceration surface, and a marking pen was used to draw the outline of the wound on the adhesive plaster. A photo of the plaster was taken using a digital camera against a white background. In ImageJ2x, the outline of the photo was outlined and the pixel area value was recorded as A. The pixel area value of the minimum of coordinate grid that came with the quick wound healing adhesive plaster in the photo was measured and recorded as B. The following Formula was used to calculate the wound area and wound closure:

$$ {\displaystyle \begin{array}{l}\mathrm{Wound}\  \mathrm{area}\ \left({\mathrm{cm}}^2\right)=\mathrm{A}/\mathrm{B}\times 0.1\ {\mathrm{cm}}^2\\ {}\%\mathrm{wound}\  \mathrm{closure}=\left[1\hbox{-} \left(\mathrm{WA}\right)/\left(\mathrm{WA}\mathrm{o}\right)\right]\times 100\\ {}\Big(\mathrm{Where}\\ {}\mathrm{WA}=\mathrm{wound}\  \mathrm{area};\\ {}\mathrm{WA}\mathrm{o}=\mathrm{original}\  \mathrm{size}\  \mathrm{of}\  \mathrm{the}\  \mathrm{wound}\  \mathrm{area}.\Big)\end{array}} $$

### Histology method to obverse re-epithelialization

For general histological analyses, tissue samples were fixed in 10% neutral-buffered formalin, embedded in paraffin, sectioned from the midline of wounds, and stained with H&E Staining.

### Reverse transcription polymerase chain reaction(RT-PCR) for the test of activin, follistatin mRNA content

On day 1, 3, 5, 7, 11, 15 after the wounding, animals were euthanized by CO_2_ and the tissues of the skin wounds were taken with an 8 mm diameter metallic punch. The tissue was put into liquid nitrogen immediately and kept at −80 °C. Extracting total RNA: The homogenate was taken and total RNA was extracted according to the Trizol reagent kit method and steps. The concentration of total RNA was measured by ultraviolet spectrophotometer. The purity of RNA was measured with agarose gel electrophoresis. Reverse transcribed to synthesize cDNA: primers used were as follows: activin forward 5’-TCGAATCTACAGGGATGAATGGA-3′, reverse 5’-GGAGGGTTTCTGGTGGGATG-3′; follistatin forward 5’-CTGAGAAAGGCCACCTGCTT-3′, reverse 5’-TCACAGGACTTTGCTTTGATACAC-3′; glyceraldehyde-3-phosphate dehydrogenase forward 5’-GGGCATCTTGGGCTACACTG-3′, reverse 5′- CATGAGGTCCACCACCCTGT-3′. Reverse Transcription System First Strand cDNA Synthesis Kit was used, with total reaction volume of 20.0 μl. Real-Time PCR amplification: ABI kit was used to make Real-Time PCR amplification of GAPDH, Activin and Follistain. Real time fluorescent PCR was used to make real-time fluorescent quantitative PCR reaction. After the reaction was finished, fluorescent quantitative data were collected and analyzed. The data included amplification curve, working curve, dissociation curve, and corresponding CT value. Light Cycler Software (Version 3.5) was used for data analysis.

### Western blot for evaluation of activin, follistatin protein expression

SJHY Formula were topically applied after punch for 15 days, diabetic vehicle and diabetic SJHY Formula mice were euthanized by CO_2_ and the tissue of the skin wounds was taken with an 8 mm diameter metallic punch. The tissue was put into liquid nitrogen immediately and kept in −80 °C. For each sample, 0.1 g was taken from the above tissue and grinded to tissue homogenate on ice(50 μL 1 × PBS was added to each sample). Then it was mixed with 50 μL 2 × cell lysis buffers and incubated for 30 min on ice. The mixture was centrifuged for 15 min, 12,000 rpm/min at 40 °C, and the supernatant was kept. Next, 50 μL for each sample was taken from the above supernatant liquid, mixed with 50 μL 2 × SDS loading buffer and heated in 100 °C water baths for 5 min. After cooling, the mixture was centrifuged for 1 min, 3000 rpm/min. Electrophoresis using the Bio-Rad EP system was done on 20 μg of each sample. The condition was 70 V for 30 min for the first time and 90 V for 100 min for the second time. The protein was transferred from polyacrylamide gel to nitrocellulose filter membrane with Bio-Rad transfer system for 2 h in ice bath, 100 V. The protein was stained in Ponceau S staining solution for 5 to 10 min then washed with water. The nitrocellulose membrane with the target protein was tailored according to molecular mass. The membrane was put in 30 ml block buffers and shaken at room temperature for 1 h on the shaking table. Primary antibody(Ab) reaction: specific antibody of relevant target protein (1:1000) was added to the solution and incubated at room temperature for 2 h on the shaking table. The membrane was washed by 1 × TBST for 3 times, once for 5 min. Secondary Ab reaction: the corresponding secondary Ab of horseradish peroxidase (HRP) coupling was added according to the proportion of 1:5000(Jackson 1:2000)and the membrane was hatched on shaking table at room temperature. The membrane was washed by 1 × TBST for 5 times, once for 15 min. ECL luminescence reaction: the membrane was dealt with enhanced chemiluminescence (ECL) luminescence reagent, developed and exposed. Odyssey quantitative fluorescence scanner was used for developing, 700(red) or 800(green) was chosen for quantitative analysis. ImageJ1.42q was used for quantitative analysis to get scan results. Meanwhile, the relative absorbance of objective strip was ensured (the absorbance of the control strip of each western blot was set as relative value 1). GAPDH has been used as Western blot loading control antibodies.

### Immunohistochemistry to investigate pSmad2 and NF-kB p50 nuclear staining

SJHY Formula were topically applied after punch for 15 days, diabetic vehicle and diabetic SJHY Formula mice were euthanized by CO_2_ and the tissues of the skin wounds were extracted using an 8 mm diameter metallic punch. The tissue was put into liquid formalin and embedded by paraffin. The formalin-embedded tissue was sliced into 6~8-μm-thickness slices. Slices were dewaxed and washed by distilled water and PBS. Formalin mixed with 3% hydrogen peroxide was used to block endogenous peroxidase for 15 min. Then the slices were washed by PBS for 3 times and fetal calf serum was used to block non-specific antigen sites for 30 min. Primary Ab was added and incubated for an hour at 37 °C. Then the slices were washed by PBS for 3 times. Secondary Ab was added and incubated for 30 min at 37 °C. Then the slices were washed by PBS for 3 times. Tissues were developed by DAB, dyed again by hematoxylin and mounted into neutral balsam. Images were taken by Olympus BH2 Upright Metallurgical Microscope.

### Statistical methods

The statistical analyses were carried out with IBM SPSS Statistics, version 21.0 package. The enumeration data were expressed as rate (%) and the measurement data were expressed as mean ± S.E.M. Multiple comparisons among groups were performed by one-way ANOVA. Comparisons in groups were performed by Tukey’s test. For uneven variance, variance was corrected and analyzed. Repeated measures data were performed by repeated measurement analysis of variance. Data with non-normal distributions were performed by non-parametric test. Statistically significant results were expressed as *p* ≤ 0.05, *p* ≤ 0.01, *p* ≤ 0.005.

## Results

### Loureirine a and loureirine B were determined by HPLC to establish the quality standard for SJHY formula

**Fig. 1:**
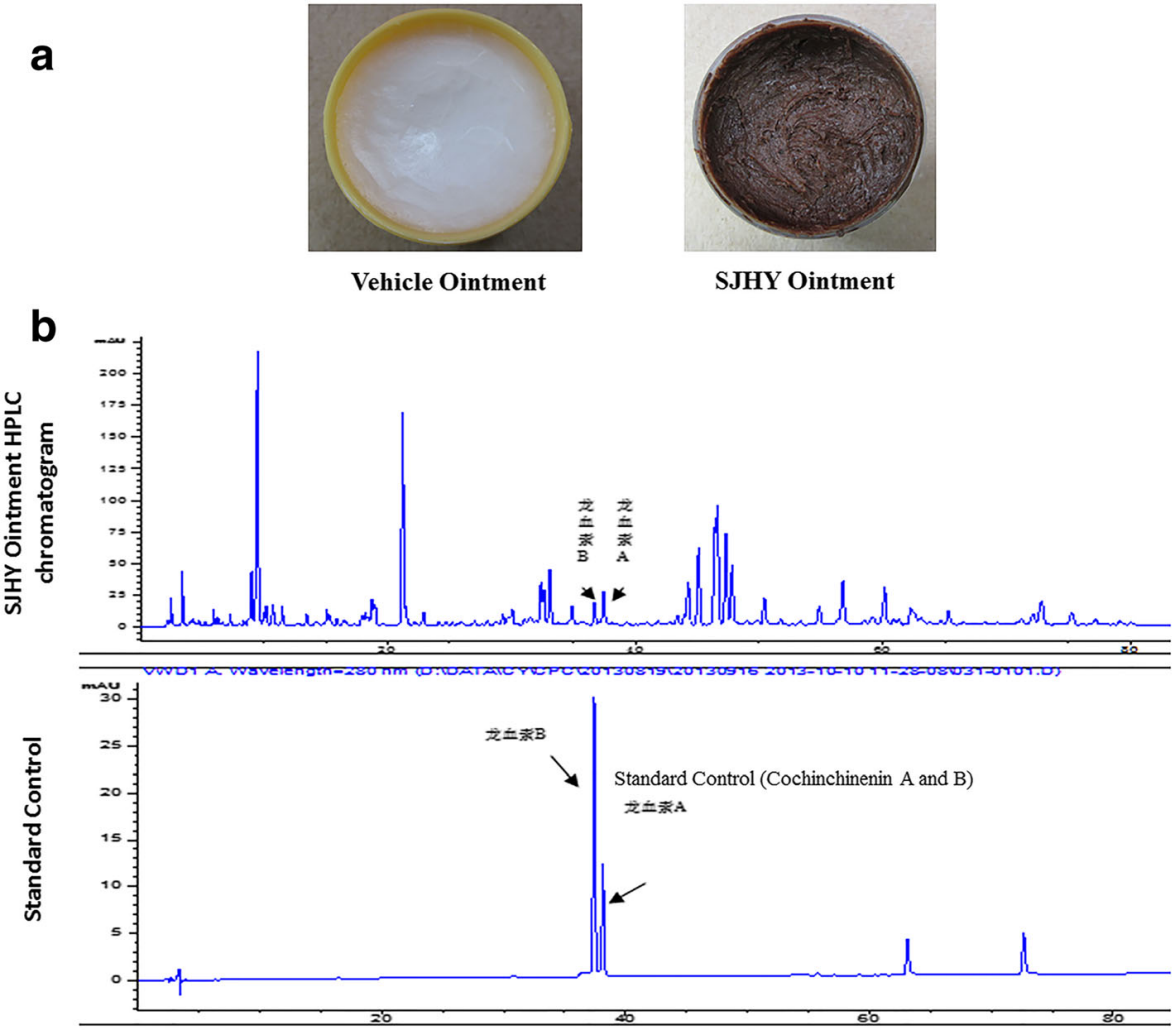
Quantitative analysis of SJHY Formula by HPLC. **a**. Presentation of Vehicle ointment (left) and SJHY ointment (right). **b**. HPLC method was used to determine the SJHY quality control, SJHY Ointment chromatogram (up panel) and standard control of loureirin A and loureirin B (lower panel)

HPLC method was used to determine the SJHY quality control, SJHY Ointment chromatogram (upper panel) and standard control of loureirin A and loureirin B (lower panel) (Fig. 1(b)).

Logarithmic equation of external standard method was used to calculate the content. The content of loureirine A in the SJHY Formula is 0.167 mg/g, RSD = 0.59%, and loureirine B in the SJHY Formula is 0.145 mg/g, RSD = 1.49% (*n* = 6) (Table. 2).

**Table 2:** Result of determination Loureirin A and B in SJHY Formula (*n* = 6)

source	Loureirin A	RSD	Loureirin B	RSD
	/mg/g	/%	/mg/g	/%
*SJHY Formula*	0.167	0.59	0.145	1.49

### SJHY formula accelerated re-epithelialization of diabetic wound healing

Histologically, epidermal migrating tongues in SJHY treated wounds were more advanced than in diabetic wound. While SJHY treated wound completely healed, diabetic wound showed unhealed wound epithelia, moreover, the epidermis in SJHY treated was significantly thinner and more regular than those in diabetic control (Fig. 4).

### Activin/follistatin mRNA levels were highly expressed during wound healing in diabetic wound

**Fig. 2:**
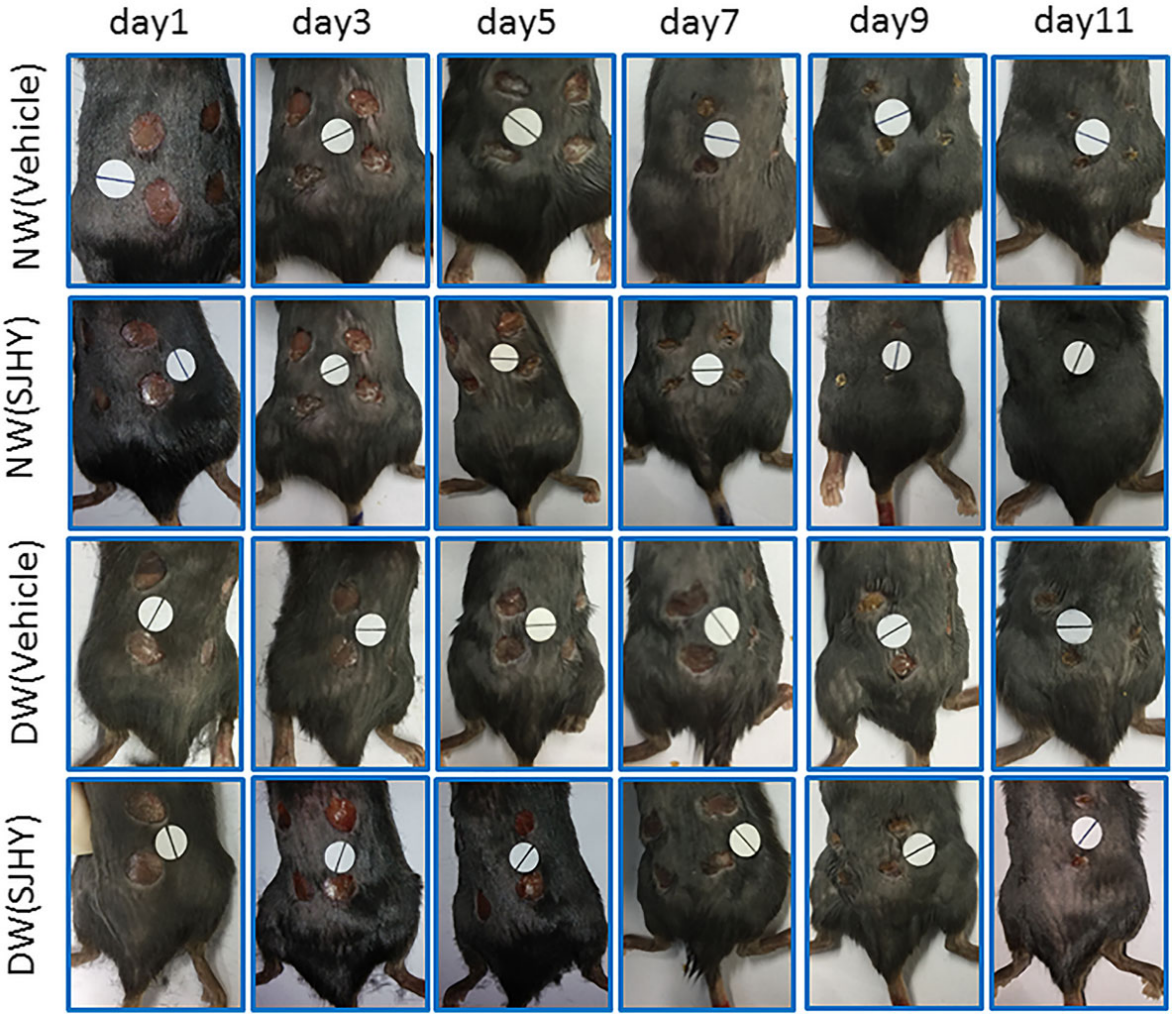
Photographic representation of wound closure on different post wounding days. 1: Diabetic SJHY Formula-treated group 2: Diabetic vehicle group (carbomer) 3: Normal SJHY Formula-treated group 4: Normal vehicle group. Wounding days starting from day1st, 3rd, 5th, 7th, 9th, 11th, day respectively

**Fig. 3:**
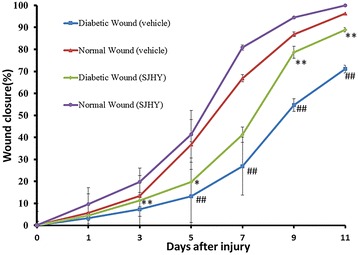
Effect of SJYH Formula treatment in normal and diabetic wound from day0 to day11 after punch. A hundred and twenty-eight wounds from thirty-two animals were analyzed at each time point. It showed diabetic wound delays wound healing and SJHY Formula promotes wound healing. Mean values ± SD are shown. Two-sample equal variance *t*-test followed by Bonferroni’s correction was used. * *p* < 0.05, ** *p* < 0.01, compared with diabetic wound. ^#^*p* < 0.05, ^##^*p* < 0.01, compared with normal wound

Diabetic ulcers were harder to heal than normal wounds. Wound closure area analysis also indicated a significant difference on day 5,7,9 and 11 after wounding (day5:13.30% ± 7.68% in diabetic wound versus 36.77% ± 9.22% in normal wound; day7:26.86% ± 15.46% in diabetic wound versus 66.88 ± 11.26% in normal wound; day9:54.71% ± 13.17% in diabetic wound versus 86.86% ± 1.67% in normal wound; day11:71.09% ± 2.85% in diabetic wound versus 96.29% ± 1.05% in normal wound, *p* < 0.01) (Fig. 2 and Fig. 3).

To evaluate the effect of activin/follistatin in diabetic wound healing, activin/follistatin mRNA expression (relative to Gapdh) from the wound around the skin tissue of normal and diabetic mice injured after day 1, 3, 5, 7, 11, 15 were examined by real time-PCR. Activin/follistatin mRNA levels were highly expressed during wound healing in diabetic wound than normal wound through day1 to day15 after wounding, with significant difference on day11, 15(day11:1.54 ± 0.30 in diabetic wound versus 0.66 ± 0.26 in normal wound; day15:1.50 ± 0.14 in diabetic wound versus 0.88 ± 0.27 in normal wound, *p* < 0.001) (Fig. 5).

### SJHY formula accelerated diabetic wound healing, reduced protein expression of activin/follistatin regulation, and reduced pSmad2, NF-kB p50 nuclear staining

SJHY Formula accelerated diabetic wound healing time. Wound closure area analysis also indicated a significant difference on day 3rd,5th,9th and 11th after wounding(day3:11.29% ± 1.54% in diabetic SJHY versus 7.30% ± 2.48% in diabetic control, *p* < 0.01; day5:19.68% ± 2.69% in diabetic SJHY versus 13.30% ± 7.68% in diabetic control, *p* < 0.05; day9:78.68% ± 3.51% in diabetic SJHY versus 54.71% ± 13.17% in diabetic control, *p* < 0.01; day11:88.92% ± 2.70% in diabetic SJHY versus 71.09% ± 2.85% in diabetic control, *p* < 0.01) (Fig. 2 and Fig. 3).

To further determine the molecular biology mechanism for SJHY Formula-treated diabetic ulcer. We detected activin and follistatin proteins by Western Blot. Protein samples from the wound around the skin tissue in diabetic SJHY (SJHY Formula were topically applied after punch for 15 days) and diabetic control. SJHY Formula considerably reduced activin/follistatin protein levels, with significant difference(0.94 ± 0.10 in diabetic SJHY versus 1.36 ± 0.17 in diabetic control, *p* < 0.05) (Fig. 6a and Fig. 6b). Additionally, reduction of pSmad2 and NF-kB p50 nuclear staining was shown in the epidermis of diabetic SJHY versus diabetic control on day15 (Fig. 7).

## Discussion

SJHY formula is one of the most efficient TCM in the treatment of the delayed diabetic wound. Studies have shown SJHY Formula increases the level of collagen types I and III in granulation tissue of rats on the seventh day of wound healing [22], while regulating their ratio. SJHY formula also could adjust their metabolism in wound healing fibroblasts [23], as well as the expression of MMP-3 and TIMP-1 in ulcer tissues of diabetic rats [24]. Yet reaction mechanism for excessive inflammation in the process of re-epithelialization due to imbalanced expression of pro-inflammatory and anti-inflammatory factors is not clear yet.

Delayed wound healing in diabetic is linked to TGF-β superfamily that many studies have focused on [9–12]. Much progress has been made in identifying the effect of activin and its antagonist follistatin in cutaneous wound healing; activin can accelerate wound healing but form excessive scar tissue [14–18], while follistatin reduces the inflammatory reactions that inhibit excessive scar formation [19]. However, the exact effect of activin/follistatin in the process of cutaneous wound healing is still not clear. Our study found SJHY Formula decreases high expression of activin/follistatin in the delayed diabetic wound healing.

**Fig. 4:**
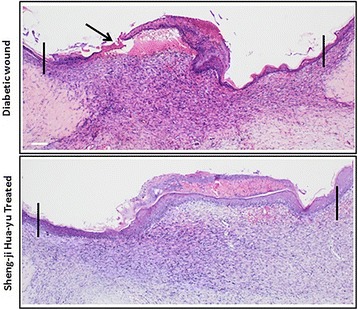
Re-epithelialization in wounded skin by H&E staining. Note allows indication in diabetic wound showed unhealed wound epithelia, while SJHY treated wound completely healed. Scale bar = 100 μm

**Fig. 5:**
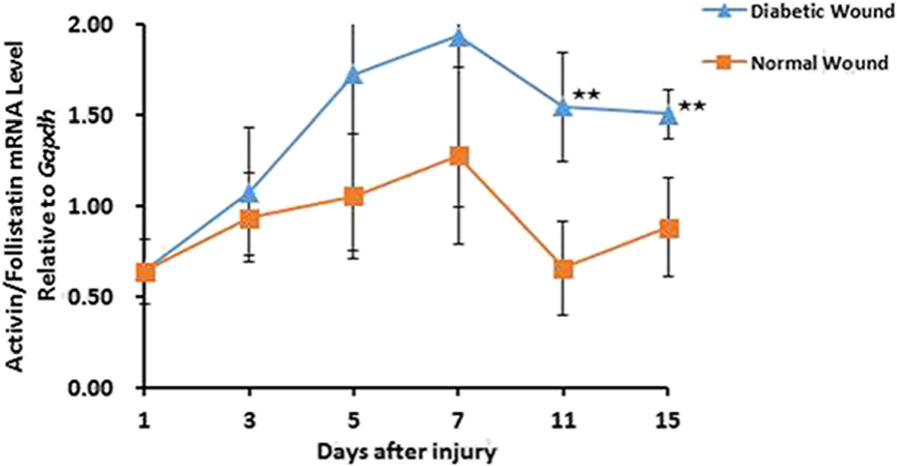
RNA samples from the skin of normal and diabetic mice injured after 1, 3, 5, 7, 11, 15 days were analyzed for expression of Activin and Follistatin (relative to Gapdh) by RT-PCR. The scale of Activin and Follistatin was shown. Bars indicate mean value ± SD. *n* = 8 mice in each time point. Two-sample equal variance *t*-test followed by Bonferroni’s correction was used. ^★^*P* < 0.05 and ^★★^*P* < 0.001 compared with normal mice

**Fig. 6:**
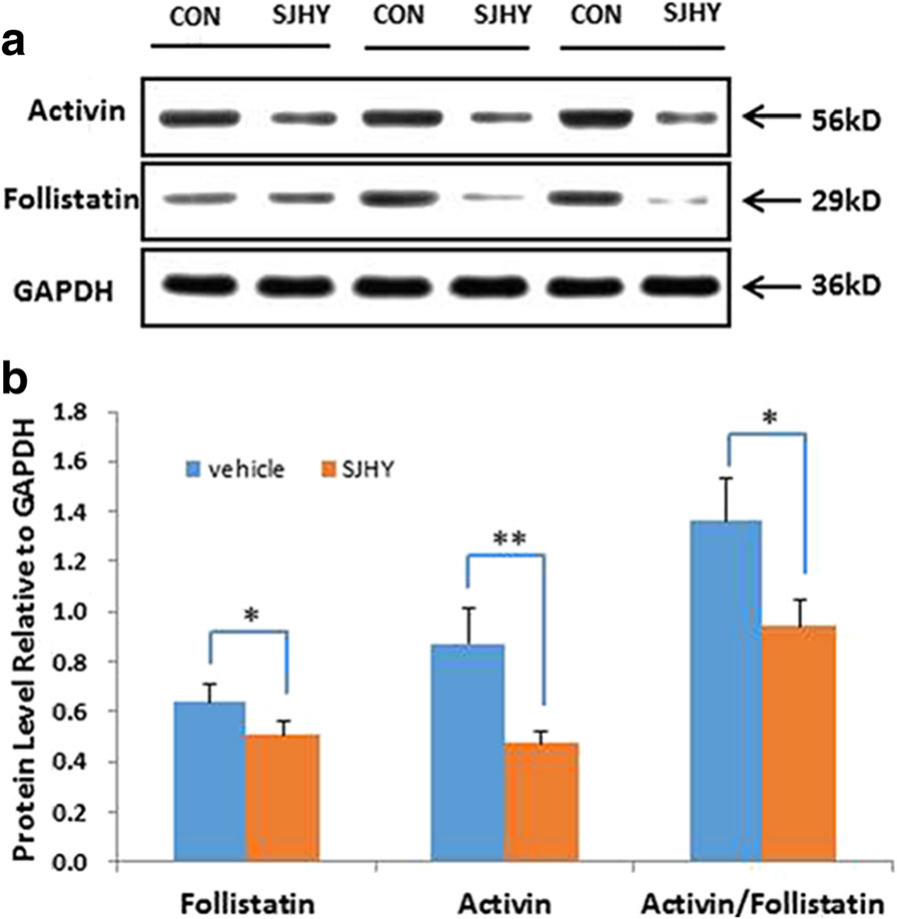
**a**: Detection of Activin and Follistatin proteins by Western Blot immunoassay. Protein samples from the skin in diabetic mice, of which vehicle and SJHY Formula were topically applied after the punch for 15 days. **b**: Activin and Follistatin protein level values of SJHY Formula and vehicle treatment in diabetic mice. Mean values ± SD are shown. *n* = 8 diabetic vehicle and 8 diabetic SJHY Formula mice. Two-sample equal variance *t*-test followed by Bonferroni’s correction was used. *P < 0.05 and **P < 0.001 vs diabetic vehicle

**Fig. 7:**
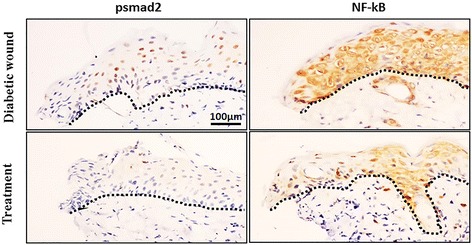
Investigating pSmad2 and NF-KB p50 nuclear staining. Protein samples from the skin in diabetic mice, of which vehicle and SJHY Formula were topically applied after the punch for 15 days. Dotted lines delineate epithelial-stromal boundary. Scale bar = 100 μm

Histological observation showed that when SJHY treated wound had completely healed, there is evident epithelial migration compared to diabetic control, signifying that SJHY Formula could accelerate re-epithelialization of diabetic wound healing (Fig. 4). Activin/follistatin system is involved and crucial for wound repair [27, 28]. Our current study has indicated that during wound healing, activin/follistatin mRNA levels were highly expressed in diabetic skin. We suggest that it might be a critical factor leading to delayed diabetic wounds. On the other hand, SJHY Formula accelerated healing time of diabetic wound from a macroscopic view, which is consistent with the former report described by Li B et al. and Wang Y F et al. [21, 23]. According to the study that activin/follistatin mRNA levels were highly expressed during wound healing in diabetic wound (Fig. 5). Western blot analysis showed that SJHY Formula has a down-regulating effect on the level of activin/follistatin (Fig. 6a and Fig. 6b). Decreasing high expression of activin/follistatin is shown to be one of the molecular biology mechanisms for SJHY Formula to treat diabetic ulcers. To further confirm whether SJHY Formula accelerates epidermal closure in diabetic wounds by regulating activin/follistatin, we examined pSmad2, a surrogate marker for activation of Smad-dependent TGF-β signaling on day 15 after wounding. Reduced pSmad2 nuclear staining in diabetic SJHY wounds was observed in the epidermis at the wound edge in comparison with diabetic control skin, which corresponds with the effect to reduce the downstream inflammatory response (Fig. 7). In addition, we examined NF-kB p50, a nonsecreted protein complex controlling transcriptional regulation of inflammatory cytokines. Nuclear translocation of NF-kB p50 protein was down-regulation in diabetic SJHY compared to diabetic control (Fig. 7), which could significantly reduce inflammatory cytokine production in keratinocytes. Therefore the molecular biology mechanism for SJHY Formula in treating diabetic ulcers can be attributed to the reduction of high expression of activin/follistatin that could result in excessive inflammation. It will be interesting to apply topical activin and follistatin treatment during wound healing in vivo and cultivate keratinocytes in vitro to explore how activin/follistatin regulation works and the effect of SJHY Formula on cell growth.

## Conclusion

The present study found that diabetic delayed wound healing time is closely related to the high expression level of activin/follistatin which leads to excessive inflammation in the process of re-epithelization. SJHY Formula accelerates re-epithelialization and healing time of diabetic wounds through decreasing the high expression of activin/follistatin.

## Abbreviations

Ab: antibody
CSU: chronic skin ulcer
ECL: enhanced chemiluminescence
ERK: extracellular regulated protein kinases
HE: Hematoxylin-eosin
HRP: horseradish peroxidase
JNK: c-Jun N-terminal kinase
LC-MS: liquid chromatograph-mass spectrometer
MMP-3: Matrix metalloprotease-3
NF-kB: Nuclear factor kappa B
NS: normal saline
PBS: Phosphate buffer saline
PDA: photodiode array
pSmad2: phospho-Smad2
RhoA: Ras homolog gene family, member A
RT-PCR: Reverse transcription polymerase chain reaction
SJHY: Sheng-ji Hua-yu
SPF: specific pathogen-free animal
Stz: Streptozotocin
TCM: Traditional Chinese Medicine
TGF-β: transforming growth factor-β
TIMP-1: Tissue inhibitor of metalloproteinase-1
WM: western medicine
Wnt: wingless/int1-related
